# Single-cell multi-omics integration for unpaired data by a siamese network with graph-based contrastive loss

**DOI:** 10.1186/s12859-022-05126-7

**Published:** 2023-01-04

**Authors:** Chaozhong Liu, Linhua Wang, Zhandong Liu

**Affiliations:** 1 Graduate Program in Quantitative and Computational Biosciences, Baylor College of Medicine, Houston, USA; 2 Jan and Dan Duncan Neurological Research Institute at Texas Children’s Hospital, Houston, USA; 3 Department of Pediatrics, Baylor College of Medicine, Houston, USA

**Keywords:** Single-cell sequencing analysis, Data integration, Deep learning, COVID-19

## Abstract

**Background:**

Single-cell omics technology is rapidly developing to measure the epigenome, genome, and transcriptome across a range of cell types. However, it is still challenging to integrate omics data from different modalities. Here, we propose a variation of the Siamese neural network framework called MinNet, which is trained to integrate multi-omics data on the single-cell resolution by using graph-based contrastive loss.

**Results:**

By training the model and testing it on several benchmark datasets, we showed its accuracy and generalizability in integrating scRNA-seq with scATAC-seq, and scRNA-seq with epitope data. Further evaluation demonstrated our model's unique ability to remove the batch effect, a common problem in actual practice. To show how the integration impacts downstream analysis, we established model-based smoothing and cis-regulatory element-inferring method and validated it with external pcHi-C evidence. Finally, we applied the framework to a COVID-19 dataset to bolster the original work with integration-based analysis, showing its necessity in single-cell multi-omics research.

**Conclusions:**

MinNet is a novel deep-learning framework for single-cell multi-omics sequencing data integration. It ranked top among other methods in benchmarking and is especially suitable for integrating datasets with batch and biological variances. With the single-cell resolution integration results, analysis of the interplay between genome and transcriptome can be done to help researchers understand their data and question.

**Supplementary Information:**

The online version contains supplementary material available at 10.1186/s12859-022-05126-7.

## Background

Diseases like cancer, heart disease, and Alzheimer's are highly complex [1]. Unlike simple Mendelian single-gene disorders, their progression is dictated by multiple genetic and environmental factors from various molecular layers, creating etiological and clinical heterogeneity that complicates diagnosis, treatment, and drug development [2]. High-throughput technologies that measure multiple omics data at the single-cell level, such as scRNA-seq [3, 4] and scATAC-seq [5, 6] have explained part of this heterogeneity from cell-type differences. However, due to unpaired cells in different omics datasets, we still lack a comprehensive and integrated view of all omics data. We therefore need to integrate different omics information to elucidate potential causative changes that lead to disease, or treatment targets, which can then be tested in further molecular studies [7].

Two main strategies have been proposed to integrate different omics data modalities: the experimental approach [8–11], which profiles multiple omics data simultaneously on the same cells, and computational approaches, which fuse independent omics datasets. With the low throughput and high cost of experimental approaches [12], the continued development of computational methods is critically important. Yet integrating multi-omics datasets remains challenging due to the unpaired cells and modality/batch effects.

The unpaired cell effect refers to the problem created when different omics data are sequenced from different batches of cells so there is no correspondence available to link modalities. To solve this problem, researchers typically project all the cells into a shared latent space, from which unpaired cells can be aligned to share all omics data. Seurat [13] applies canonical correlation analysis [14] (CCA) to project datasets into this space and aligns cells by mutual nearest neighbors (MNN) for data fusion and label transfer. But the use of a linear dimension reduction algorithm has been criticized as it will distort the actual interrelationships between datasets [15]. This linearity assumption was also adopted by Liger [16], which uses integrated non-negative matrix factorization [17]. Deep learning offers an alternative method for nonlinear projection using an autoencoder [18]’s encoder module, which projects high-dimensional data into a low-dimensional representation with one or several layers of neurons. This method has been applied successfully in GLUE [19] using a variational autoencoder.

The second challenge arises from both modality and batch effects during integration. Most algorithms remove modality effect when projecting and aligning cells but to our knowledge, batch effect is not especially considered in these integration models. The Siamese neural network [20] has been shown to integrate multiple scRNA-seq datasets and remove batch effects [21], and we believe this framework can also be used to integrate multi-omics data while eliminating both modality and batch effects. However, it was trained to integrate single-modality RNA-sequencing data at a cell type level rather than perform the multi-omics integration task.

Therefore, we introduce here a new Siamese neural network design with a graph-based loss to integrate multi-omics datasets at single-cell resolution. Trained to integrate cells from different modalities while removing the potential batch effect, our model outperforms other algorithms in multiple benchmarking datasets. Furthermore, to show the integration’s impact on downstream analysis, we developed a model-based smoothing and cis-regulatory element-inferring approach and demonstrated its efficacy by validating in 10X Multiome datasets. Finally, we applied the framework and analysis to a published COVID-19 dataset, improving the original work by adding integrated, multi-modal analysis.

## Results

### Integrating single-cell multi-omics data through the MinNet framework

As with other state-of-the-art integration methods, the MinNet framework follows the statistical concept of integrating omics data: Cells from different modalities are projected into the same latent space that captures the shared variance in all omics data. To generate this co-embedding space, the Siamese neural network simultaneously receives as inputs one cell from modality 1 (e.g., scRNA-seq) and another from modality 2 (e.g., scATAC-seq) and projects them into the same n-dimensional vectors using the encoder. To ensure this n-dimensional vector space is a good representation of the shared main biological variance, two losses are applied following the encoder.

The first and most important is the contrastive loss [22]. Here, we convert the concept of shared main variance to a more computationally feasible metric for the neural network – similarity and differences among cells. An ideal co-embedding space should be consistent with the original data on this metric: similar cells are close and very different cells are far away. Thus, our contrastive loss aims to reduce the distance between similar cells and separate different cells in the n-dimensional space. To achieve this goal, randomly chosen cell pairs are prepared before each training epoch for calculating either positive or negative contrastive loss. Positive pairs are the identical cells in the two modalities, and the loss is the Euclidian distance between each pair in the co-embedding spaces. Negative pairs are different cells sampled from the data, and the loss is calculated as a margin constant $m$ minus the Euclidian distance. By training the model to minimize the loss, the distances between corresponding cells get smaller while the distances between negative pairs get larger. In this way, main biological variance is kept in the co-embedding space.

**Fig. 1:**
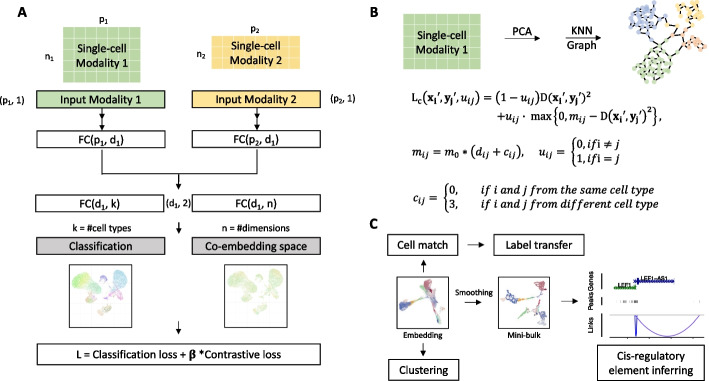
Overview of MinNet. **A** Model receives two modalities’ data as input. High-throughput omics data will go through an independent fully connected layer to be projected into a lower dimensional space. This representation space should be able to mix different modalities and separate cell types well. To achieve this, cell type classification loss and Siamese contrastive loss are used during the training process. **B** To make the mixing resolution at single-cell level rather than cell-type level, we applied a KNN graph-based Siamese loss with flexible margin value depending on cell pair graph distance. **C** In application, multiple omics data will be projected into this low-dimensional embedding space in which downstream analysis will be done, including cell alignment, label transfer, unsupervised clustering, and the designed cis-regulatory element-inferring pipeline

Usually, the margin value *m* is a constant for all negative pairs during Siamese neural network training. However, cells from different cell types are more diverse than those from the same cell type. Thus, to maintain these differences in the co-embedding space, we designed *m*as a flexible value depending on how much the cell pairs differ. (See Methods for technical details). Intuitively, cells that differ more pose higher variance, so a larger margin is assigned to separate them in the space. In contrast, similar cells pose little variance, so a small margin value is assigned (Fig. 1B). With the flexible margin, the datasets main variance will be better kept in the final integration space.

The second loss is cell-type classification loss. It is also designed to capture the main variance because it separates different cell types from each other. The output of the first encoder layer is sent to the label classification layer for cross-entropy loss calculation. Also, previous studies observed improved performance with this loss due to its ability to accelerate the optimization process [21].

This supervised model needs to be trained with paired multi-omics datasets from techniques like 10X Multiome, SHARE-seq [9], and SNARE-seq [8], which profile the transcriptome and chromatin accessibility simultaneously, or Cite-seq [23], which profiles transcriptome and epitopes in the same cells. The weighted sum of classification and contrastive loss is minimized during training to ensure optimized modality mixing and clustering. After training, the model can be easily applied in user’s target datasets.

We applied the framework to two tasks: transcriptome and chromatin accessibility data integration, which takes gene expression and gene activity score as its input; and transcriptome and epitope data integration taking gene expression and protein abundance. With the trained models, users can provide two simply normalized datasets and obtain the co-embedding space for downstream analysis, including aligning/pairing cells between modalities, unsupervised clustering, and cis-regulatory element inferring via pseudo-bulk generated from the embedding space (Fig. 1C).

### Benchmarking shows that MinNet is robust and generalizable in alignment and clustering

To test the performance and generalizability of our two models trained on 10X Multiome bone marrow mononuclear cells (BMMC) data and Cite-seq BMMC data, we evaluated our method and compared it with existing ones, including GLUE [19], bindSC [24], Seurat v3 [13], Liger [16], and Liger's online version [25], on two untouched test sets from the NeurIPS 2021 Competition [26] in which the cell-to-cell correspondence is known. This large dataset has 10X Multiome and Cite-seq sequencing results from four sequencing sites and ten donors. Our models were trained on samples from some of the donors and three sequencing sites, leaving other donors untouched (test set 1) and the fourth sequencing site untouched (test set 2) (See Additional file 1: Table S1 for details).

**Fig. 2:**
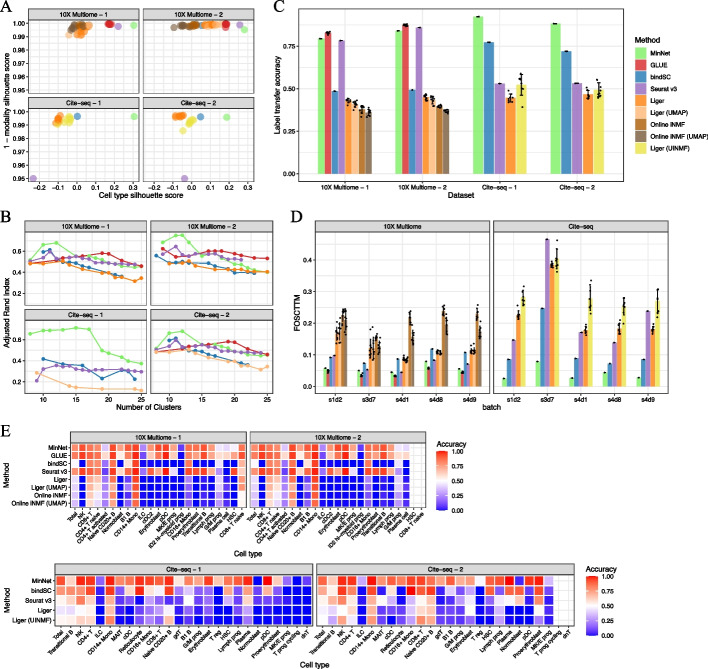
Performance benchmarks on gold-standard datasets. To test our model and compare it to existing algorithms, we benchmarked the transcriptome and chromatin accessibility data integration model and the transcriptome and cell-surface protein data integration model on datasets from the NeurIPS 2021 competition data. **A** Silhouette scores on the embedding space generated by all algorithms. Cell type silhouette score indicates how well cell types separate from each other, and 1– modality silhouette score indicates how well modalities mix with each other. **B** Adjusted Rand index along with the number of clusters comparing all algorithms. **C** Average label transfer accuracy bar plot. **D** FOSCTTM (Fraction of samples closer than the true match) score indicates the single-cell level alignment error of all algorithms. **E** Label transfer accuracy heatmap from transcriptome data to chromatin accessibility data (top); or from epitope data to transcriptome data (bottom)

After applying all algorithms to the benchmarking datasets, we evaluated all final integration results (see Additional file 1: Figs. S1–4 for the UMAP visualization) based on several metrics. First, we used the silhouette coefficient score [27] to measure the integration performance of the co-embedding space generated by the algorithms, focusing on how well modalities are mixing while cell types are separating from each other. Compared with other methods, MinNet attained a higher score in both modality mixing and clustering (Fig. 2A). The cell type silhouette coefficient indicates how well cell types are separated in the co-embedding space. In the real-world setting, when researchers have no labels for their dataset, they will use unsupervised clustering to annotate the cell types; a better separation will ensure more precise annotations. We tested this proposition by performing unsupervised clustering on the algorithms' embeddings and testing the consistency between unsupervised clusters and cell type annotations using the adjusted rand index [28] (Fig. 2B). Results show that MinNet-based clustering is the most concordant with the ground truth at the primary cell type level. Moreover, when subtypes were identified, our model was still competitive with the top models in 10X Multiome data and outperformed all models in Cite-seq data. We also evaluated cell type integration performance based on label transferring accuracy, another common task in actual practice when researchers want to transfer the annotated labels from one modality to the other. MinNet can accurately transfer most of the labels, even if the cell numbers are small, while methods like Seurat are biased toward the major cell types (Fig. 2C, E). With Silhouette score and label transfer accuracy, our model’s performance is validated at the cell type level.

Beyond the cell type resolution integration, single-cell level cell alignment is also essential in some cases, like cell type sub-typing and mini-bulk generation for downstream analysis. To evaluate this higher resolution performance, the FOSCTTM (Fraction of samples closer than the true match) score [29] was measured for all generated co-embeddings. MinNet ranked in first or second place in all four datasets, and performed especially well in Cite-seq data where it demonstrated a significant competitive advantage at single-cell resolution (Fig. 2D).

For broad usability, a supervised model must be generalizable. Our model’s success in integrating untouched donor datasets and untouched sequencing site datasets already demonstrated its generalizability, but we also wanted to test it with other tissues. First, we undertook the same evaluation using the 10X Multiome peripheral blood mononuclear cell (PBMC) dataset [30]. Based on silhouette scores, FOSCTTM scores, and label transfer accuracies (Additional file 1: Fig. S5A, C, D), our model still performed competitively and generated adequate co-embedding space (Additional file 1: Fig. S6). Though the model had never seen many of the cell types in the PBMC dataset, it still separated most cell types well, demonstrating its generalizability. This result is due to the model’s contrastive loss design, which learned the common sense of similar tasks rather than a specific task [31].

However, this generalizability was limited to similar tissues, such as BMMC and PBMC. We also applied the trained model to the 10X Multiome human brain dataset [32], an entirely different tissue. The resulting co-embedding space showed little biological information and failed to cluster well (Additional file 1: Fig. S5B). Therefore, we concluded that the generalizability of our algorithm could be expanded to similar tissues but not distinct ones. Nevertheless, this supervised approach can easily be trained on target tissues and has a higher specificity than other models. For example, the traditional machine learning models, including bindSC, Liger, and Seurat, have better label transfer accuracies on the PBMC dataset than on the BMMC dataset, which we believe is due to cell type balance. That is, when the numbers of cells in each cell type are relatively even, these methods perform well. But in cases like the BMMC datasets, which consist mostly of monocytes, label transfer of minor cell types is inaccurate. In contrast, our approach is not significantly influenced by unevenly distributed cell type sizes because of its superior specificity.

### MinNet is superior in removing batch effect while maintaining biological variance

To distinguish between batch variance and biological variance, we trained our model with multiple batches from different donors and sequencing sites. While the training input was normalized data without batch correction, the contrastive loss was based on the graph after batch correction by ComBat implemented in Scanpy [33]. With this design, the model is required to produce the joint embedding that eliminates batch effects while retaining biological differences.

To test the performance of batch effect removal, we generated three more testing scenarios with the available benchmark datasets that represent real case practice problems. The first scenario tested all algorithms’ performance when both scRNA-seq and scATAC-seq experiments are performed independently on identical batches. The second and third scenarios tested the integration performance of scRNA-seq and scATAC-seq datasets profiled from different batches. In all three cases, we compared our model with those mentioned above.

**Fig. 3:**
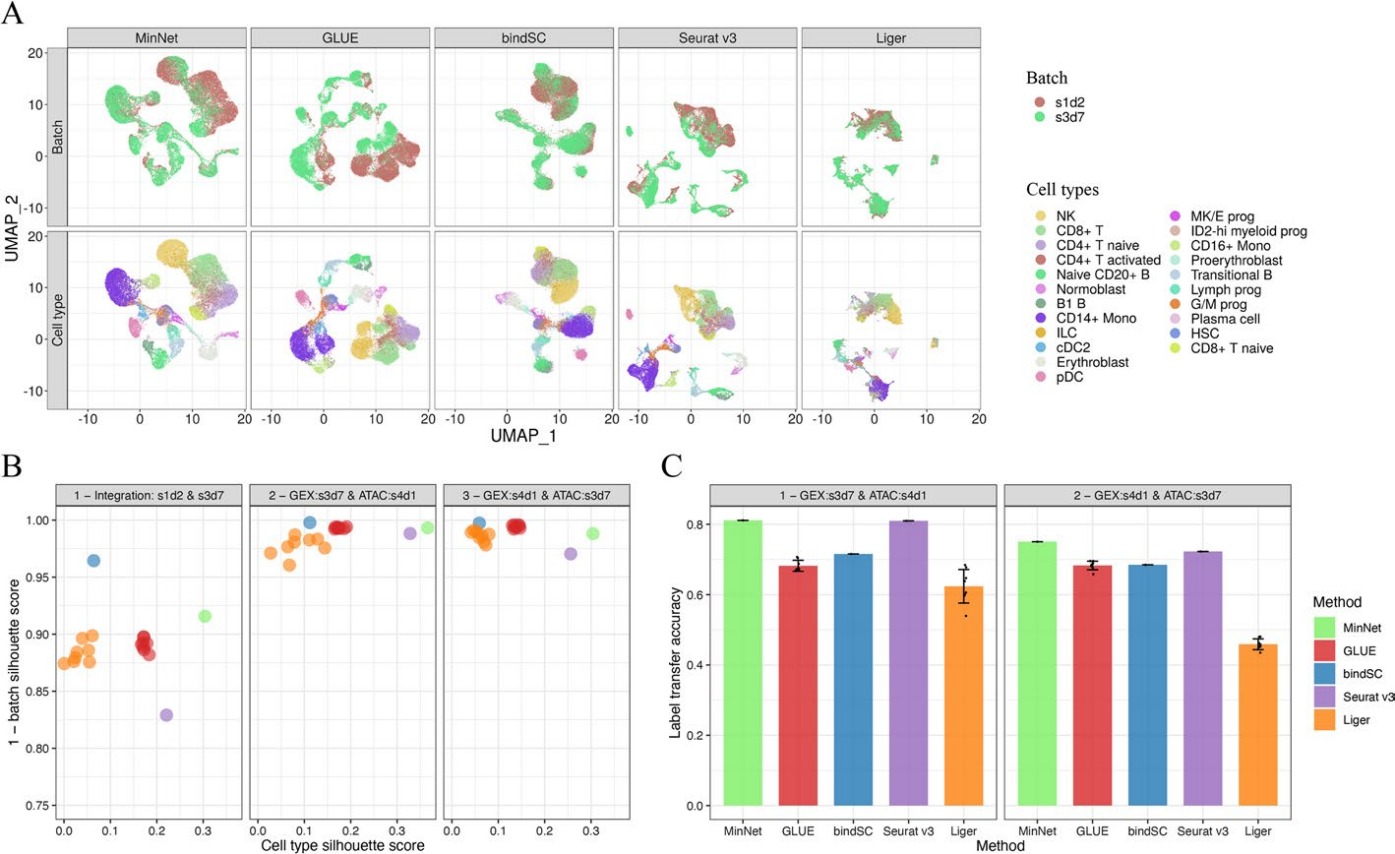
MinNet batch effect removal outperforms other algorithms. While separating cell types and mixing modalities, our model showed the best performance in removing batch effect, the most common challenge in integrating different omics data from distinct sources. **A** UMAP visualization of the embedding space generated by all algorithms. **B** Silhouette score indicates that while separating cell types, our model mixes batches well. **C** Label transfer accuracy from one donor’s transcriptome data to another donor’s chromatin accessibility data

The silhouette score and label transfer accuracy were chosen for evaluation since cells were different in the second and third cases and FOSCTTM score is not feasible. Figure 3A shows the co-embedding space of the first case. While mixing the omics data, MinNet successfully separated cell types and mixed the batches. Its performance is quantified and compared with other algorithms in Fig. 3B using cell type and batch silhouette scores. BindSC was better at mixing batches, but MinNet distinguished the cell type variance from batch variance to provide better cell type separation. In the last two tests, most algorithms generated a co-embedding space that mixed the batch well, meaning all the manifold alignments worked between two single-batch omics data (Additional file 1: Figs. S7, S8). However, when it came to the mixed batches, some algorithms failed due to their inability to remove the batch effect, resulting in small cell type silhouette scores (Fig. 3B). MinNet thus outperformed the other models in clustering and label transferring (Fig. 3C).

This evaluation is especially important as it mimics real-world practice in which researchers use independent profiling from different batches or even from independent, publicly available datasets. We also conducted testing on Cite-seq data in a similar case setting (Additional file 1: Fig. S3) and observed that none of the algorithms succeeded in mixing batches from different donors and sequencing sites, but they could mix batches from other donors and the same sequencing sites (Additional file 1: Fig. S4). We hypothesize that this result is due to the significant sequencing platform differences of Cite-seq technology and believe further investigation is warranted.

### Model-based smoothing helps correlation-based cis-regulatory element inferring

After benchmarking our model, we demonstrated how integration can impact the downstream analysis and help discover the interplays among genetic layers.

Cis-regulatory elements, such as enhancers and promoters, are genomic regions that control development and physiology by regulating gene expression [34]. Inferring the regulation between open chromatin regions and gene expression is of great importance in understanding biological and disease processes. Usually, cis-regulatory element inferring is done by calculating the correlation between chromatin regions, e.g., Cicero [35]. With multi-omics data and integration methods available, we can calculate the correlation between regions and gene expression using the aligned cells, which is a more direct way of linking genome with transcriptome. Here, we implemented smoothing, mini-bulk generating, and cis-regulatory element inferring and validated the method using the 10X Multiome peripheral blood mononuclear cells (PBMC) dataset [30].

**Fig. 4:**
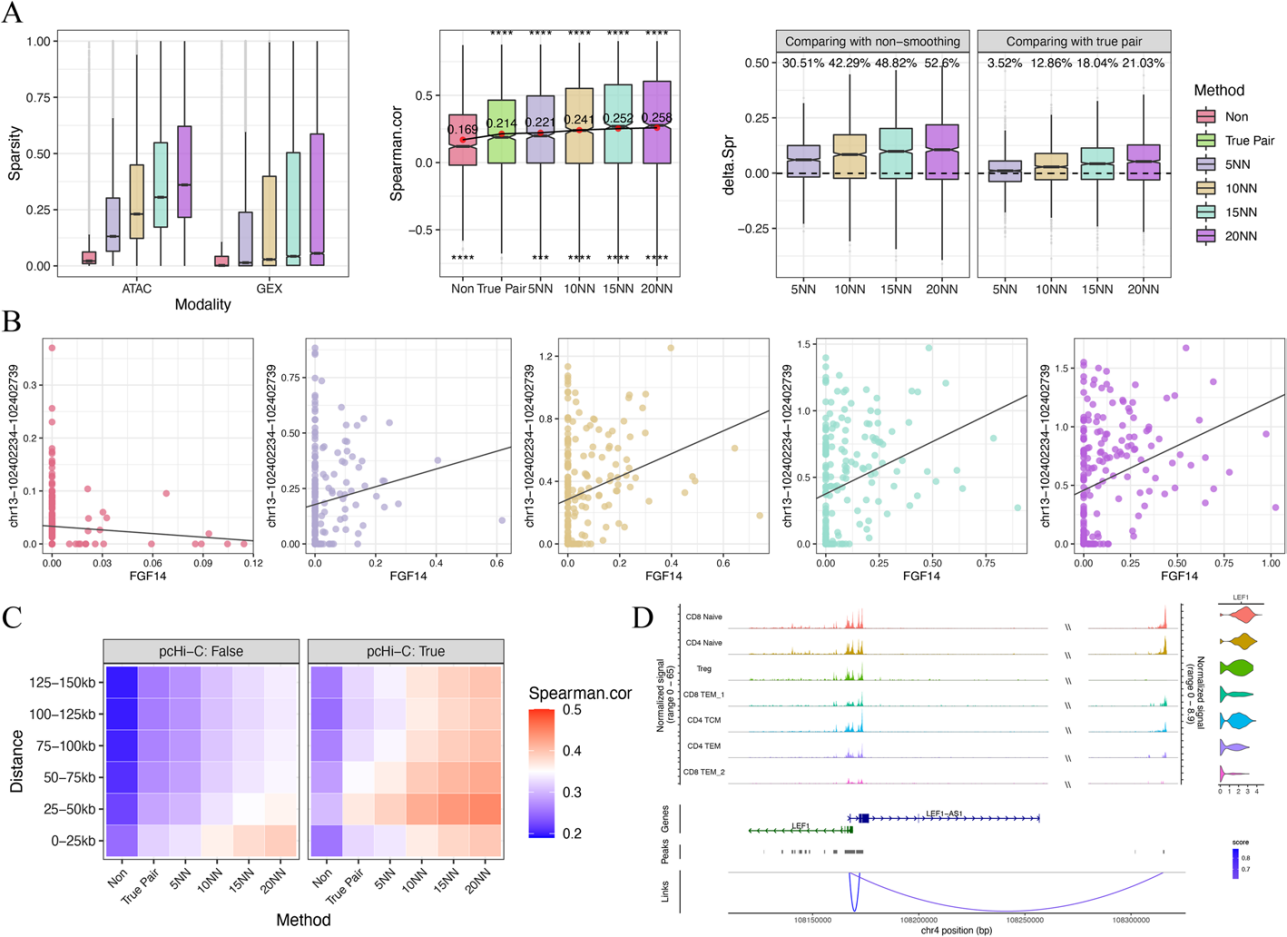
Model-based smoothing and cis-regulatory element inferring. By smoothing and generating mini-bulk omics profiles summing up neighborhood cells, we can infer the gene regulatory regions by calculating the correlation between transcriptome and chromatin openness. A higher correlation indicates a likely regulatory relationship between genes and peaks. **A** (Left) Smoothing decreased the sparsity of scRNA-seq and scATAC-seq data. (Middle) Smoothing increased the correlation between gene expression and its TSS regions openness compared with non-smoothed and true pair derived mini-bulk data. (Right) This trend is emphasized when showing the Spearman correlation coefficient differences between smoothed and non-smoothed mini-bulk data. **B** Example showing FGF14 and its TSS region peaks correlation in non-smoothed and smoothed data. **C** Heatmap showing the mean of correlation level between gene-peak pairs with different distances in all smoothed and non-smoothed datasets. **D** Genome track of LEF1 and its highly correlated peaks. Left shows the genome tracks of ATAC-seq data, right violin plots show the gene expression level

First, to account for the high dropout rate and noise [36] in single-cell data, we built functions to smooth [37] the data. Specifically, we complemented the missing values in cells based on their K nearest neighbors in our single-cell resolution co-embedding space to decrease the sparsity (Fig. 4A). After smoothing, we generated mini-bulk data before undertaking any downstream analysis.

To test how this smoothing and mini-bulk generating improve downstream analysis, we calculated the Spearman's correlations between genes and their 2 kb nearby peaks in mini-bulk data, which are believed to be positively correlated. Results show that non-smoothed raw mini-bulk data has a lower correlation level than true pair mini-bulk data correlation, meaning the dropout rate compromises downstream analysis when no cell correspondence is available between modalities (Fig. 4A middle). But when smoothing was applied to the five nearest neighbors, the correlation levels reached that of the true pair mini-bulk. The correlation was even higher than the true pair when the number of neighbors was increased. To demonstrate the importance of smoothing, we offer an example in Fig. 4B. Chr3:102402234–102402739 is in the TSS region of the gene FGF14, which means the pair should be positively correlated. But because of the high dropout rate, non-smoothed mini-bulk data showed a negative Spearman correlation coefficient. When we applied nearest neighbor complementation, their association became positive.

We further validated the model-based, cis-regulatory element-inferring approach with external Promoter Capture Hi-C (pcHi-C) evidence of the interaction between genome regions [38]. We applied non-smoothed, true pair, and smoothed mini-bulk data to calculate Spearman’s correlation between genes and their 150 kb nearby peaks. While the mean correlations of pcHi-C unsupported peak-gene pairs were not greatly increased by smoothing, the mean correlation level of pcHi-C supported pairs did increase. The difference in correlation between supported and unsupported pairs is clearly shown in the heatmap (Fig. 4C). Again, with only five nearest neighbors smoothing, the correlation reached the same level as true pair mini-bulk (Figure S9A), but the 0-25 k peak-gene pairs are non-distinguishable. We think the proximity of genes increased the co-openness even though they don't have a regulatory relationship.

The extremely high correlation peak-gene pairs yielded by this approach are worth further investigation because they indicate potential regulatory relationships. For example, LEF1 encodes the protein that can bind to a functionally important site in the T-cell receptor-alpha enhancer [39] and therefore shows a variant expression level in subtypes of T cells. Three peaks are within the 150 kb upstream of the LEF1 TSS region. In 5NN smoothing mini-bulk correlation, two peaks have high correlations with LEF1 expression (chr4-108170508–108173850: ${r}_{s}$ = 0.930; chr4-108315129–108315649: ${r}_{s}$ = 0.877) and are supported by pcHi-C evidence. The other has a low correlation and is not supported by pcHi-C (chr4-108301923–108302013: ${r}_{s}$= 0.371). These results showed consistency with Hi-C and are validated by the data visualized in Fig. 4D. On the other hand, some unsupported correlations also showed potential regulatory relationships. CCR2 encoded protein is a receptor for monocyte chemoattractant protein-1, a chemokine that specifically mediates monocyte chemotaxis [40, 41]. It is a monocyte marker; thus, the expression varies in many PBMC cell types. Six peaks showed high correlation with CCR2, three were supported by Hi-C evidence (chr3-46206526–46210451: ${r}_{s}$ = 0.647; chr3-46297386–46301922: ${r}_{s}$ = 0.612; chr3-46212,074–46213996: ${r}_{s}$ = 0.612) and three were not (chr3-46317953–46318717:${r}_{s}$ = 0.634; chr3-46312405–46313554: ${r}_{s}$ = 0.607; chr3-46228191–46229079: ${r}_{s}$ = 0.605). But when validated in the original data, we saw a correlation of all six peaks with the gene, indicating potential genomic links (Additional file 1: Additional file 1: Fig. S9B). Furthermore, the unsupported chr3-46312405–46313554, together with Hi-C supported chr3-46,206,526–46,210,451 and chr3-46297386–46301922, were enriched in the motif for STAT3 + IL-21 binding, providing further evidence supporting this finding. IL-21 is a known cytokine with diverse effects on immune cells, including CD4 + and CD8 + T cells, B cells, macrophages, monocytes, and dendritic cells [42]. Thus, CCR2 might be involved in IL-21-induced cell adhesion through these binding sites, which has been considered by other researchers [43].

### MinNet provides missing analysis of COVID-19 multi-modal data

To demonstrate how our model can help study diseases, we applied it to a publicly available COVID-19 dataset [44] where healthy controls and patients with various Worldwide Health Organization (WHO) severity score-rated PBMC samples were profiled with independent scRNA-seq and scATAC-seq. We followed their scRNA-seq Differential Expressed Genes (DEGs) analysis using the PBMC trained- and BMMC trained-models and provided the missing part of the integration analysis to allow for more potential discoveries.

**Fig. 5:**
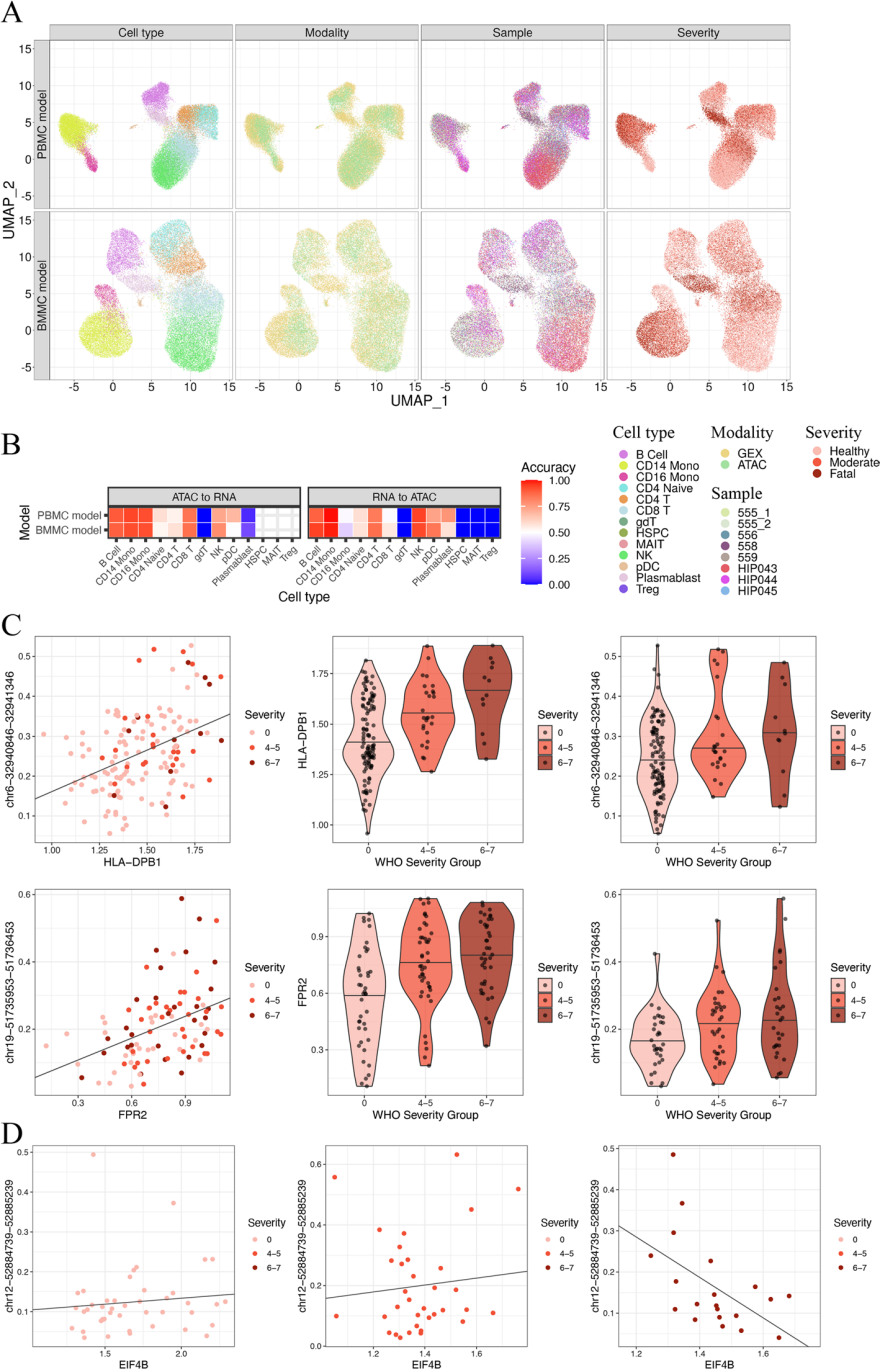
Application to COVID-19 dataset discovered cell type specific changes. Two trained models (BMMC and PBMC) were applied to this COVID-19 dataset. Healthy volunteers’ and patients’ PBMC transcriptome and chromatin accessibility were profiled independently to study immune system changes based on the severity of COVID-19 infection. **A** UMAP visualization of the COVID-19 dataset labeled by cell type, modality, sample ID, and severity. **B** Label transfer accuracy indicates that our embedding is consistent with the original cell type annotation on the majority cell types. Cell types with bad accuracy are due to only a few cells. **C** Example of regulatory element inferring from NK cells (upper) and monocytes (lower). **D** Dysfunction of EIF4B may be due to the change in the regulatory role of correlated open regions

Preprocessed and normalized data were provided to either the PBMC or BMMC trained model to create the final co-embedding space (Fig. 5A). The final integration space separated cell types and mixed modalities well. The batch effect from different samples was removed, while the difference in severity was still clearly apparent, as shown in the UMAP colored by WHO severity score. We next evaluated the consistency between the cell type annotation and our clustering by calculating label transfer accuracy from scATAC-seq to scRNA-seq or the reverse direction (Fig. 5B). Except for cell types with only a few cells, the consistency was high between the two independent annotations and our embedding space. Both the PBMC and BMMC models were able to integrate the dataset because of their generalizability.

Using the model’s co-embedding space, we generated the mini-bulk data per cell type and then inferred the potential regulatory relationships between genes and peaks within a 150 k bp distance from the transcription starting point. Two cell types, Natural Killer (NK) cells and monocytes, were chosen for the integration analysis because they were the most dysfunctional cell types identified in the original study. Only DEGs by severity group were included in this analysis as compensation for their primary scRNA-seq DEG analysis. For example, in NK cells, the DEG HLA-DPB1 is correlated with chr6-32940846–32941346 (${r}_{s}$ = 0.333, Fig. 5C, Additional file 1: Fig. S10A) and showed differences among WHO severity groups. In monocytes, the DEG FPR2 is associated with the peak chr19-51735953–51736453 (${r}_{s}$ = 0.434, Fig. 5C, Additional file 1: Fig. S10B). These results were further validated with the raw data and could be potential regulatory sites for the DEGs.

Besides inferring the causal relationships between COVID-19 influenced peaks and genes, we can also compare the correlations among severity groups to discover dysfunction in severely infected groups. Thus, we generated the pseudo-bulk data per cell type for each severity group separately and calculated Spearman’s correlation between genes and peaks within a 150 k bp distance. The inconsistency in correlation level might indicate the dysfunction in regulation between genes and peaks. For example, in monocytes, EIF4B and its remote peaks chr12-52884739–52885239 are positively correlated in healthy control and moderately infected patients but are negatively correlated in severely infected patients (Fig. 5D). Such findings indicate that the peak's positive regulation is destroyed in severely infected patients. One potential rationale is that the enhancing protein was competitively replaced by another inhibitory protein during the enhancer and promoter interaction, leading to the negative correlation between openness and expression.

## Discussion

We constructed this single-cell resolution multi-omics data integration model by designing the flexible margin contrastive loss based on the graph's shortest distance. We successfully applied it to human BMMC scRNA-seq, scATAC-seq, and epitope data integration. In benchmarking its performance, our model ranked among the top existing algorithms based on silhouette score, FOSCTTM score, and label transfer accuracy. Since it can be trained in multiple batches and in loss design, the model can distinguish batch variation from actual biological variation and generate a better co-embedding space while mixing batches well. With the single-cell resolution and batch effect-removed embedding, better pseudo-bulk data can be generated for correlation-based cis-regulatory element inferring in integrating scRNA-seq and scATAC-seq. Using a real COVID-19 dataset, this model showed how it can fill the gap in current multi-omics data analysis.

In designing the model, we also tried using more complicated architecture, including convolutional layers and multi-head attention layers [45]. A previous transcriptome prediction model demonstrated the success of the attention mechanism [46], so we tried using peaks as scATAC-seq input and multi-head attention to extract the low-dimensional representation. After training, the attention model performed quite well, but its generality and performance were not as good as our final fully-connected neural network which is also light and fast. Theoretically, complex models with more parameters are prone to overfit the training data, especially when the cell numbers are limited in our case. In a standard single-cell RNA-seq analysis pipeline, Principal Component Analysis can capture the main variance decently with only linear transformation. Thus, we think fully-connected layers with linear transformation and activation function have enough complexity to solve the integration problem while maintaining the generality. Nevertheless, it would be worth experimenting with an attention-based model using larger sample sizes and computational capacity in the future.

Our supervised model does have obvious drawbacks. Although we demonstrated its generalizability by showing that our BMMC trained model can be successfully applied to different donors, sequencing sites, and even PBMC tissues, application to entirely different tissues still requires additional training on the specific tissue. However, we argue that this additional training is easily achievable. First, the number of paired single-cell multi-omics data is growing, providing sufficient tissue- and organism-specific training samples. Second, only a few hyper-parameters, including margin altitude ${m}_{0}$, learning rate $r$, and weight of contrastive loss $\lambda$, need to be tuned. Lastly, the training process is standardized and easily executable. But although applying the algorithm to a different dataset is easy, we are still working on more generalizable and unsupervised multi-modal integration models.

We also plan to generalize our two-omics integration framework to multiple omics integration. This is beneficial when researchers have more than two sets of omics data in hand for integration analysis. This aim is achievable because there are merging techniques like scNMT-seq [10] and scTrio-seq [47] that measure three omics modalities, which can be used as training datasets. By generalizing the Siamese pair generation and training process to any number of omics data, the MinNet framework is capable of performing more than two omics integration if the training data is available.

Another future improvement involves the gene activity score. This transformation of peaks is known to lose information [48] and algorithms like GLUE and bindSC therefore perform integration while optimizing the feature transformation between peaks and genes. Maintaining peak information makes the model more accurate, so we will consider using this design to improve our current MinNet model.

## Conclusions

MinNet is a novel deep-learning framework for single-cell multi-omics sequencing data integration. With our graph-based flexible margin contrastive loss, it reached single-cell resolution integration and ranked top among publicly available methods in benchmarking. Moreover, with special attention to batch effect, MinNet poses the unique ability in distinguishing batch and biological variances as compared to other methods. With our simplified and standardized training process, users can easily train their model to achieve high specificity with respect to the research organisms or tissues. With MinNet and model-based cis-regulatory element inferring, users can explore the potential causal interplays between epigenome and transcriptome, as we demonstrated in the COVID-19 study. Finally, MinNet offers a novel and feasible framework to solve integration problem with the Siamese neural network.

## Methods

### The Siamese neural network

The model simultaneously receives as inputs one cell from the single-cell modality 1 and another from the single-cell modality 2, denoted as $\mathbf{x} \in {\mathbb{R}}^{1\times {p}_{1}},$ $\mathbf{y} \in {\mathbb{R}}^{1\times {p}_{2}}$. ${p}_{1}$is the number of features in modality 1, and ${p}_{2}$ is the number of features in 2. The *x* and *y* will go through the encoding module first to get ${\mathbf{x}}^{\mathbf{^{\prime}}},\mathbf{y}\mathbf{^{\prime}} \in {\mathbb{R}}^{1\times h}$, $h$ is the number of units in the hidden layer. Then, ${\mathbf{x}}^{\mathbf{^{\prime}}},\mathbf{y}\mathbf{^{\prime}} \in {\mathbb{R}}^{1\times h}$ are linearly transformed into ${\mathbf{x}}_{\mathbf{p}\mathbf{r}\mathbf{e}\mathbf{d}},{\mathbf{y}}_{\mathbf{p}\mathbf{r}\mathbf{e}\mathbf{d}} \in {\mathbb{R}}^{1\times k}$ vectors representing the probability of cells belonging to each of the $k$ cell types. Cross entropy loss is used for the final classification loss ${L}_{l}$:

$${\text{L}}_{{\text{l}}} \left( {{\mathbf{x}}_{{{\mathbf{pred}}}} ,labelx} \right) = - \mathop \sum \limits_{c = 1}^{k} {\text{I}}\left( {c,labelx} \right) \cdot \log \frac{{\exp \left( {x_{pred,c} } \right)}}{{\mathop \sum \nolimits_{i = 1}^{k} \exp \left( {x_{pred,i} } \right)}},$$

$${\text{I}}\left( {c, labelx} \right) = \left\{ {\begin{array}{*{20}c} {0,} & {if} & {c \ne labelx} \\ {1,} & {if} & {c = labelx} \\ \end{array} } \right.$$

where $labelx$ stands for the cell type label. ${\mathrm{L}}_{\mathrm{l}}({\mathbf{y}}_{\mathbf{p}\mathbf{r}\mathbf{e}\mathbf{d}},labely)$ is defined in the same way. Meanwhile, ${\mathbf{x}}^{\mathbf{^{\prime}}},\mathbf{y}\mathbf{^{\prime}} \in {\mathbb{R}}^{1\times h}$ is linearly transformed to${\mathbb{R}}^{1\times 32}$ vectors representing its position on the final 32-dim joint embedding space. The contrastive loss is calculated as follows:

$${\text{L}}_{{\text{c}}} \left( {{\mathbf{x}}^{\prime},{\mathbf{y}}^{\prime},u} \right) = \left( {1 - u} \right) \cdot {\text{D}}\left( {{\mathbf{x}}^{\prime},{\mathbf{y}}^{\prime}} \right)^{2} + u \cdot {\text{ max}}\left\{ {0, m - {\text{D}}\left( {{\mathbf{x}}^{\prime},{\mathbf{y}}^{\prime}} \right)^{2} } \right\},$$

$u$ is the label indicating whether the two cells are corresponding pairs ($u$=0) or not ($u$=1). $\mathrm{D}(\bullet )$defines the distance between ${\mathbf{x}}^{\mathbf{^{\prime}}}$ and ${\mathbf{y}}^{\mathbf{^{\prime}}}$. *m* here is the margin predefined between each pair of different cells using the shortest distance between the two in a KNN graph generated in the preprocessing step (explained next). Intuitively, cells far away from each other in the graph have larger margin values; highly similar cells that are close in the graph have smaller margin values (Figure 1B). Thus, the total loss is:

$${\text{L}}\left( {{\mathbf{x}},{\mathbf{y}}} \right) = {\text{L}}_{{\text{l}}} \left( {{\mathbf{x}}_{{{\mathbf{pred}}}} ,labelx} \right) + {\text{L}}_{{\text{l}}} \left( {{\mathbf{y}}_{{{\mathbf{pred}}}} ,labely} \right) + \lambda \times {\text{L}}_{c} ,$$

The is the weight between classification loss and contrastive loss.

### Determining the flexible margin from KNN graph

During training data processing, one of the omics data is processed with batch correction, principal component decomposition (PCA), and KNN graph construction. The modality chosen is scRNA-seq for 10X Multiome and Cite-seq training data, because it presents the variation in data better in most cases. With the graph, the shortest distance ${d}_{ij}$between all cell pairs is calculated as part of the margin ${m}_{ij}$. The contrastive loss margin ${m}_{ij}$ of cell $i$ from modality 1 and cell $j$ from modality 2 is defined as:

$$m_{ij} = m_{0} *\left( {d_{ij} + c_{ij} } \right), c_{ij} = \left\{ {\begin{array}{*{20}c} {0, } & { if\; i \;and\; j \;from\; the \;same \;cell\; type} \\ {3,} & { \;if \;i \;and \;j \;from \;different \;cell\; type} \\ \end{array} } \right.$$

${m}_{0}$ is a constant controlling the scale of contrastive loss and can be the tunable hyper-parameter. $c$is used to increase the penalty of two cells of different cell types being close to each other in the co-embedding space. Value 3.0 worked in all our scenarios.

### Training process

The two preprocessed feature matrices are scaled by genes to unit variance and zero mean, followed by clipping values larger than 10. We use the Adam optimizer to train the model with user-provided hyper-parameter values including ${m}_{0}$ QUOTE, $\lambda$, and learning rate. Before each epoch, the two matrices are shuffled, and all cells are randomly assigned either a positive (same cell in different modalities) or negative (different cells) cell pair to calculate the contrastive loss. The number of negative pairs and positive pairs is controlled near 3:1.

Two assigning strategies were tried and performed equally well. The first is the between-modality strategy, in which negative pairs are different cells in different modalities. The second is the within-modality strategy, in which negative pairs are different cells in the same modality. Because the positive pairs are the same for the two strategies, both within and between-modality co-embedding space correction works well. In the final model, we chose the between-modality strategy for the scRNA-seq and scATAC-seq integration tasks, and the within-modality strategy for the scRNA-seq and cell surface protein integration tasks.

### Data processing

The preprocessed and well-annotated bone marrow mononuclear cells data from the NeurIPS 2021 competition can be downloaded in GSE194122. The AnnData object was loaded in Python 3.6.13 with AnnData 0.7.6. All Scanpy-based processing mentioned below is done with Scanpy 1.7.2.

### NeurIPS 2021 competition 10X multiome training data

Samples from sequencing sites 1, 2 and 3 were taken as the training dataset, including s1d1, s1s3, s2d1, s2d4, s2d5, s3d3, s3d6, and s3d10. S stands for sequencing site and d stands for donor number. First, we performed feature and cell selection. Highly variable genes in scRNA-seq data of all batches were determined by Scanpy *pp.highly_variable_genes* function with Cell Ranger flavor. Only genes marked as highly variable genes in more than one batch were kept. We also kept cell surface protein genes as the features for training. We then performed stricter cell filtering based on mitochondria gene expression proportion (< 4), number of genes expressed (100–4000), number of peaks (1000–80,000), and the total number of fragments (1000–300,000). This is the final feature set and cell set for training.

We used the already processed gene activity matrix saved in the Anndata obsm *gene_activity*. It is the count sum of peaks 2 kb upstream of the selected genes' TSS region, calculated by Seurat v3. Together with the feature-selected scRNA-seq data, log-transformed per cell normalization was performed to correct sequencing depth difference. These were the final input of two matrices for model training.

To determine the margin value between cell pairs, we constructed a KNN graph using the scRNA-seq data. ComBat implemented in Scanpy was used to perform batch correction, followed by PCA and K nearest neighbor graph construction saved as a large sparse matrix in the AnnData object named *connectivity*. Distances between neighbor cells were then estimated by 1.01—*connectivity* value. To calculate the shortest distance between all pairs from the large sparse matrix efficiently, Scipy 1.5.4 *dijkstra* function was used to generate the $n\times n$ matrix recording all shortest distances for training.

### NeurIPS 2021 competition cite-seq training data

Samples from sequencing sites 1, 2 and 3 were taken as the training dataset, including s1d1, s1d3, s2d1, s2d4, s2d5, s3d1, and s3d6. We performed similar feature and cell selection as 10X Multiome data in GEX data. Highly variable genes in scRNA-seq data of all batches were determined by Scanpy *pp.highly_variable_genes* function with Cell Ranger flavor. Only genes marked as highly variable genes in more than two batches were kept. We also kept cell surface protein genes as the features for training. We then performed stricter cell filtering based on mitochondria gene expression proportion (< 15), the number of genes expressed (75–1200), and the number of peaks (75–1500). All features in ADT data were kept and cell orders were consistent between modalities. The final feature and cell-selected matrices were under log-transformed normalization before training.

The same strategy was applied as the 10X Multiome dataset to determine the shortest distances between all cell pairs.

### NeurIPS 2021 competition 10X Multiome test data

Samples from s1d2 and s3d7 were taken as the first testing set. Samples from s4d1, s4d8, and s4d9 were taken as the second testing set. Log-transformed transcriptome matrix was used as one of the inputs for the trained model. The gene activity matrix was from the already processed samples saved in the Anndata obsm *gene_activity*. Before applying the neural network, we selected the features in training and compensated for missing features with all 0 values. Then the two matrices were scaled by genes to unit variance and zero mean, followed by clipping values larger than 10. Finally, the test mode of the trained model was run to generate the 32-d co-embedding space coordinate for every cell.

### NeurIPS 2021 competition Cite-seq test data

Samples from s1d2 and s3d7 were taken as the first testing set. Samples from s4d1, s4d8, and s4d9 were taken as the second testing set. The same process was done as the 10X Multiome dataset, but we used the ADT data instead of the gene activity matrix.

### Human peripheral blood mononuclear cells (PBMCs) Multiome data from 10X Genomics

The dataset can be downloaded on 10X Genomics website at https://support.10xgenomics.com/single-cell-multiome-atac-gex/datasets/1.0.0/pbmc_granulocyte_sorted_10k. We followed all the same processing of Seurat integration tutorial document at https://satijalab.org/seurat/articles/atacseq_integration_vignette.html. The gene activity matrix was calculated using Signac 1.1.1 summing up counts 2 kb upstream of the gene TSS region. Gene activity matrix and genes count matrix were saved as HDF5 files together with the metadata. Then the files were loaded in Python and underwent log-transformed normalization using Scanpy. The subsequent processes were the same as those applied to the NeurIPS 10X Multiome data.

### Human brain multiome data from 10X genomics

The dataset can be downloaded from the 10X Genomics website at https://www.10xgenomics.com/resources/datasets/frozen-human-healthy-brain-tissue-3-k-1-standard-2-0-0. Preprocessing was done by filtering cells in RNA-seq that had less than 1000 counts, larger than 25,000 counts or high mitochondria proportion (> 10%), and filtering cells in ATAC-seq with fragment counts less than 5000 or larger than QUOTE. Only cells remaining in both modalities were kept. Dimension reduction and clustering were done following the Surat default pipeline. The gene activity matrix was calculated using Seurat v3 summing up counts 2 kb upstream of the gene TSS region. The gene activity matrix and genes count matrix were saved as HDF5 files together with the metadata. Then the files were loaded in Python and underwent log-transformed normalization using Scanpy. The subsequent processes were the same as those applied to the NeurIPS 10X Multiome data.

### JEM COVID-19 multi-omics profiling scRNA-seq data

The fully processed scRNA-seq AnnData H5AD file can be downloaded at https://www.covid19cellatlas.org/index.patient.html. Metadata is downloaded at their GitHub page at https://github.com/ajwilk/COVID_scMultiome. We performed log-transform normalization with the processed data. To stay consistent with scATAC-seq data, we kept only shared donor batches and re-annotated cell types.

### JEM COVID-19 multi-omics profiling scATAC-seq data

The raw data can be downloaded at GSE174072. The fragment files were processed using ArchR 1.0.1 following the same quality control as mentioned in the paper. The batch-specific TSS enrichment score and the minimum number of fragments cutoff can be found on their GitHub page mentioned above. It is worth mentioning that although these researchers claim the sequencing reads were aligned with the hg19 reference genome, we found that using only hg38 can yield the correct TSS enrichment score. Thus, hg38 was used in all following related processes. We followed the same data processing pipeline in the paper, removing doublets, clustering, batch correction with Harmony, and calling peaks with MACS2. To follow the same practice as the training dataset, we used the Seurat 3.1.1 *CreateGeneActivityMatrix* function to generate the gene activity matrix instead of using the ArchR-provided gene activity matrix. The saved HDF5 file was loaded in Python and compiled into AnnData object together with the metadata from their GitHub page and went through log-transformed normalization. Again, to stay consistent with scRNA-seq data, the cell type was re-annotated and only shared batches were kept. Finally, the two log-transformed matrices provided to the model followed the same pipeline as other test datasets. Summary statistics of all datasets mentioned above are available in Additional file 2: Table S2.

### Running of all algorithms

GLUE 0.1.1, bindSC 1.0.0, Seurat 3.1.1, UnionCom 0.2.3, Liger 1.0.0, its Online-iNMF and UINMF version were all included to obtain a systematic benchmarking. Due to the memory outflow problem, UnionCom failed to get the results with GLUE-provided codes on their GitHub page. All others were implemented successfully according to the authors' tutorial. All codes are available at GitHub.

We followed GLUE's tutorial at https://scglue.readthedocs.io/en/latest/tutorials.html with all default settings. We started from raw test data to run the data preprocessing and model training steps. The final cell co-embedding space was saved for all benchmarking. GLUE was run eight times with different random seeds.

We followed bindSC's tutorial at https://htmlpreview.github.io/?https://github.com/KChen-lab/bindSC/blob/master/vignettes/CITE-seq/CITE_seq.html for Cite-seq data integration and https://htmlpreview.github.io/?https://github.com/KChen-lab/bindSC/blob/master/vignettes/method_eval/method_eval.A549.html for 10X Multiome data integration task. The final embedding used was the bi-CCA generated results. BindSC was run eight times with different random seeds.

We followed Seurat's tutorial at https://satijalab.org/seurat/articles/atacseq_integration_vignette.html for both Cite-seq and 10X Multiome data integration. The final embedding space is the UMAP dimensional reduction space following Seurat integration pipeline.

Liger and its online iNMF version were implemented for 10X Multiome data integration, following the tutorials at https://htmlpreview.github.io/?https://github.com/welch-lab/liger/blob/master/vignettes/walkthrough_rna_atac.html and http://htmlpreview.github.io/?https://github.com/welch-lab/liger/blob/master/vignettes/online_iNMF_tutorial.html. Cite-seq data was integrated using Liger and its UINMF version at http://htmlpreview.github.io/?https://github.com/welch-lab/liger/blob/master/vignettes/UINMF_vignette.html. Each model was run eight times with different random seeds.

All the UMAP visualizations were done either using the software’s available functions or Scanpy default settings.

### Benchmark criteria

Silhouette score was used to evaluate how well cell types were clustered and modalities were mixed. It is a measure of how similar an object is to its own cluster (cohesion) compared to other clusters (separation). The silhouette ranges from − 1 to + 1, where a high value indicates that the object is well matched to its own cluster and poorly matched to neighboring clusters. The silhouette score was calculated using Scikit-learn 0.24.2. To measure how well cell types were clustered, we used the raw silhouette score value. For modality mixing and batch mixing, $1 - silhouette value$ QUOTE was used, i.e., the higher the score, the better the performance.

Rand index is a measure of similarity between two sets of data clustering. Adjusted rand index was calculated using Sklearn 1.0.1 *adjusted_rand_score* function comparing unsupervised clustering and cell type annotations. Unsupervised clustering was done using Scanpy *tl.leiden* function with different resolutions so that all algorithms received the evaluation on cluster numbers from eight to the number of cell types in each dataset.

FOSCTTM (Fraction of samples closer than the true match) score was used to evaluate the co-embedding space at single-cell resolution. Assuming two single-cell omics data profiled the same set of *n* cells, when cells are projected into the co-embedding space, the FOSCTTM we calculated was defined as:

$${\text{FOSCTTM}} = \frac{1}{n}\mathop \sum \limits_{i = 1}^{n} n_{2}^{i} ,$$

where ${n}_{2}^{i}$ means the number of cells in the second modality that are closer to the ${i}^{th}$cell in the first modality than its true matches in modality 2.

Label transfer accuracy is used to measure the performance of all co-embeddings on this common task. The transfer is measured from scRNA-seq cell type annotations to either scATAC-seq or cell surface protein data. While Seurat used its own label transfer method, all other algorithms' label transfer is done by weighted K nearest neighbors. That is, the label of cell from the second modality is predicted as the max weighted vote of its K nearest cells in scRNA-seq. K is chosen for each algorithm when it reached the best performance. With the predicted cell type label and the true label, the label transfer accuracy is defined as:

$$\mathrm{Accuracy}= \frac{1}{n}\sum_{i=1}^{n}\mathrm{I}({x}_{pred}^{i}, {labelx}^{i})$$

### Data smoothing, mini-bulk synthesis, and cis-regulatory element inference

Correlation-based regulatory element inferring is always weakened because of the high dropout rate. To solve this, we first undertook data smoothing and generated transcriptome and chromatin accessibility mini-bulk data with the single-cell resolution co-embedding space.

#### Smoothing

A nearest neighbor graph was constructed based on the model generated co-embedding space using Scanpy *pp.neighbors* function with default parameters and different number of neighbors, including 5, 10, 15 and 20. The raw count matrix was multiplied by the binarized connectivity matrix to complement missing values by neighbors. The connectivity matrix was binarized by two steps: (1) cells are the nearest neighbors of the target cell; (2) the distance between them should be smaller than the 95% distance percentile value. Cells passing the criteria were used to complement the target missing values by keeping the value as 1 in the binary connectivity matrix.

#### Mini-bulk

We used Scipy 1.5.3 *pdist* function to perform hierarchical clustering of all cells in the two modalities. Then with the hierarchical order and cell types, cells were cut into N = 100 mini-bulks, and each mini-bulk was ensured to contain only one cell type. The mini-bulk matrix was generated with scglue 0.1.1 *aggregate_obs* function.

Next, genes of interest were selected and their correlation with their 150 kb upstream peaks were calculated from the mini-bulk data. The abnormally high correlations between remote peaks and genes might indicate cis-regulatory relationships. Correlation results were visualized with box and violin plots using ggplot2 in R.

### pcHi-C data processing

pcHi-C data is available at https://ars.els-cdn.com/content/image/1-s2.0-S0092867416313228-mmc4.zip and https://osf.io/e594p/. Our codes were based on GLUE's processing and scglue functions but simplified to only extract the pcHi-C evident pairs. Only evidence of overlapped cell types was chosen to be validated. Then scglue was used to map these peak-gene pairs to the 10X Multiome PBMC dataset peak-gene pairs. The evidence was saved as.graphml file for reading and writing efficiency.

### Enrichment analysis with Homer

Peaks of interest were listed in BED format as the input for Homer v4.11.1. Function *findMotifsGenome* were applied to enrich the peaks of interest in known motifs. In some cases, we had only a few peaks, so the statistical test was not reliable. In such cases, we only trusted the information regarding which motifs were matched with most of the peaks.

### Data visualization

All visualization figures were done using ggplot2. The example genome tracks were plotted with ArchR *plotBrowserTrack* and Seurat v3 *CoveragePlot* function.

## Supplementary Information

**Additional file 1**: **Table S1**. The NeurIPS 2021 competition dataset summary. **Figure S1**. 10X Multiome BMMC test set 1 UMAP visualization of benchmarking algorithms. **Figure S2**. 10X Multiome BMMC test set 2 UMAP visualization of benchmarking algorithms. **Figure S3**. Cite-Seq BMMC test set 1 UMAP visualization of benchmarking algorithms. **Figure S4**. Cite-Seq BMMC test set 2 UMAP visualization of benchmarking algorithms. **Figure S5**. Model Generalizability Evaluation in external datasets. **Figure S6**. 10X Multiome PBMC dataset UMAP visualization of the co- embedding space labeled by cell type, modality, and batch. **Figure S7**. UMAP visualization of the co-embedding space labeled by cell type and batch in batch effect removal scenario 2. **Figure S8**. UMAP visualization of the co-embedding space labeled by cell type and batch in batch effect removal scenario 3. **Figure S9**. Cis-regulatory element inferring supplementary figures. **Figure S10**. Genome tracks of examples mentioned in COVID-19 data analysis.

**Additional file 2**: **Table S2**. Summary of datasets used in the study.

## Data Availability

The NeurIPS 2021 Competition data are available at GEO by GSE194122. The 10X Multiome PBMC data are available at 10X Genomics website (https://support.10xgenomics.com/single-cell-multiome-atac-gex/datasets/1.0.0/pbmc_granulocyte_sorted_10k). The 10X human brain data are available at 10X Genomics website (https://www.10xgenomics.com/resources/datasets/frozen-human-healthy-brain-tissue-3-k-1-standard-2-0-0). pcHi-C data is available at https://ars.els-cdn.com/content/image/1-s2.0-S0092867416313228-mmc4.zip and https://osf.io/e594p/. The COVID-19 data we analyzed can be downloaded following the instruction at https://github.com/ajwilk/COVID_scMultiome. MinNet is publicly available on GitHub (https://github.com/ChaozhongLiu/MinNet).

## Abbreviations

CCA: Canonical correlation analysis
MNN: Mutual nearest neighbors
BMMC: Bone marrow mononuclear cell
PBMC: Peripheral blood mononuclear cell
UMAP: Uniform Manifold Approximation and Projection
FOSCTTM: Fraction of samples closer than the true match
Hi-C: High throughput chromosome conformation capture
COVID-19: Coronavirus Disease 2019
WHO: Worldwide health organization
NK: Natural killer (cells)
DEG: Differentially expressed genes
