# Correction: Single-cell multi-omics integration for unpaired data by a siamese network with graph-based contrastive loss

**DOI:** 10.1186/s12859-023-05249-5

**Published:** 2023-03-29

**Authors:** Chaozhong Liu, Linhua Wang, Zhandong Liu

**Affiliations:** 1 Graduate Program in Quantitative and Computational Biosciences, Baylor College of Medicine, Houston, USA; 2 Jan and Dan Duncan Neurological Research Institute at Texas Children’s Hospital, Houston, USA; 3 Department of Pediatrics, Baylor College of Medicine, Houston, USA

**Correction : BMC Bioinformatics (2023) 24:5**

**https://doi.org/10.1186/s12859-022-05126-7**

Following publication of the original article [1], the authors identified an error in Fig. 2. The correct figure is given below.

**Fig. 2:**
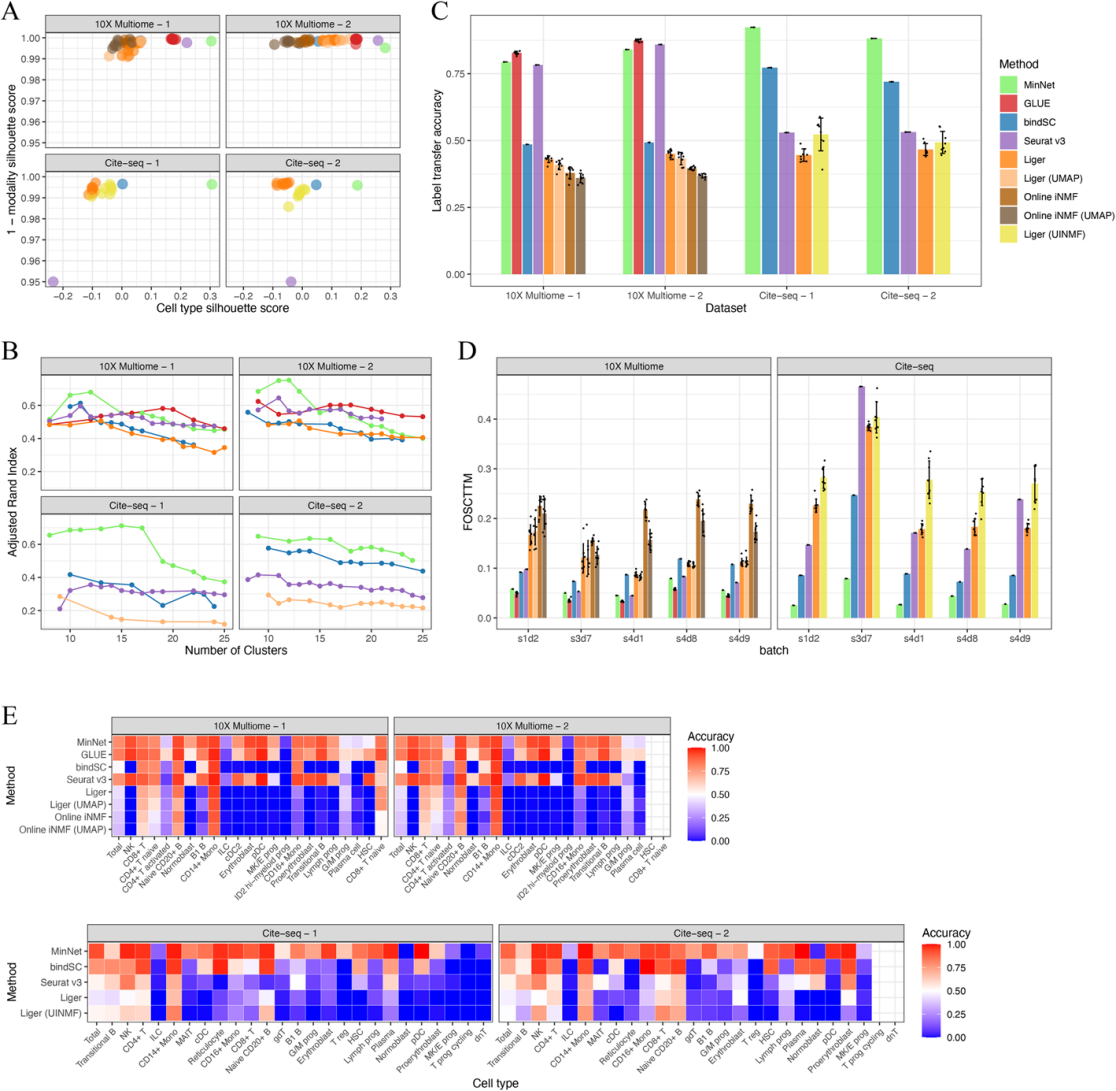
Performance benchmarks on gold-standard datasets. To test our model and compare it to existing algorithms, we benchmarked the transcriptome and chromatin accessibility data integration model and the transcriptome and cell-surface protein data integration model on datasets from the NeurIPS 2021 competition data. **A** Silhouette scores on the embedding space generated by all algorithms. Cell type silhouette score indicates how well cell types separate from each other, and 1– modality silhouette score indicates how well modalities mix with each other. **B** Adjusted Rand index along with the number of clusters comparing all algorithms. **C** Average label transfer accuracy bar plot. **D** FOSCTTM (Fraction of samples closer than the true match) score indicates the single-cell level alignment error of all algorithms. **E** Label transfer accuracy heatmap from transcriptome data to chromatin accessibility data (top); or from epitope data to transcriptome data (bottom)

The original article [1] has been corrected.
