# Supplementary material for: Single-cell multi-omics integration for unpaired data by a siamese network with graph-based contrastive loss
Source: BMC Bioinformatics. 2023 Jan 4;24:5. doi: 10.1186/s12859-022-05126-7 (PMC9812356; doi:10.1186/s12859-022-05126-7)
Supplement: Supplementary file 1 — Additional file 1: Table S1. The NeurIPS 2021 competition dataset summary. Figure S1. 10X Multiome BMMC test set 1 UMAP visualization of benchmarking algorithms. Figure S2. 10X Multiome BMMC test set 2 UMAP visualization of benchmarking algorithms. Figure S3. Cite-Seq BMMC test set 1 UMAP visualization of benchmarking algorithms. Figure S4. Cite-Seq BMMC test set 2 UMAP visualization of benchmarking algorithms. Figure S5. Model Generalizability Evaluation in external datasets. Figure S6. 10X Multiome PBMC dataset UMAP visualization of the co- embedding space labeled by cell type, modality, and batch. Figure S7. UMAP visualization of the co-embedding space labeled by cell type and batch in batch effect removal scenario 2. Figure S8. UMAP visualization of the co-embedding space labeled by cell type and batch in batch effect removal scenario 3. Figure S9. Cis-regulatory element inferring supplementary figures. Figure S10. Genome tracks of examples mentioned in COVID-19 data analysis. [file 12859_2022_5126_MOESM1_ESM.pdf]

Table S1. The NeurIPS 2021 competition dataset summary.

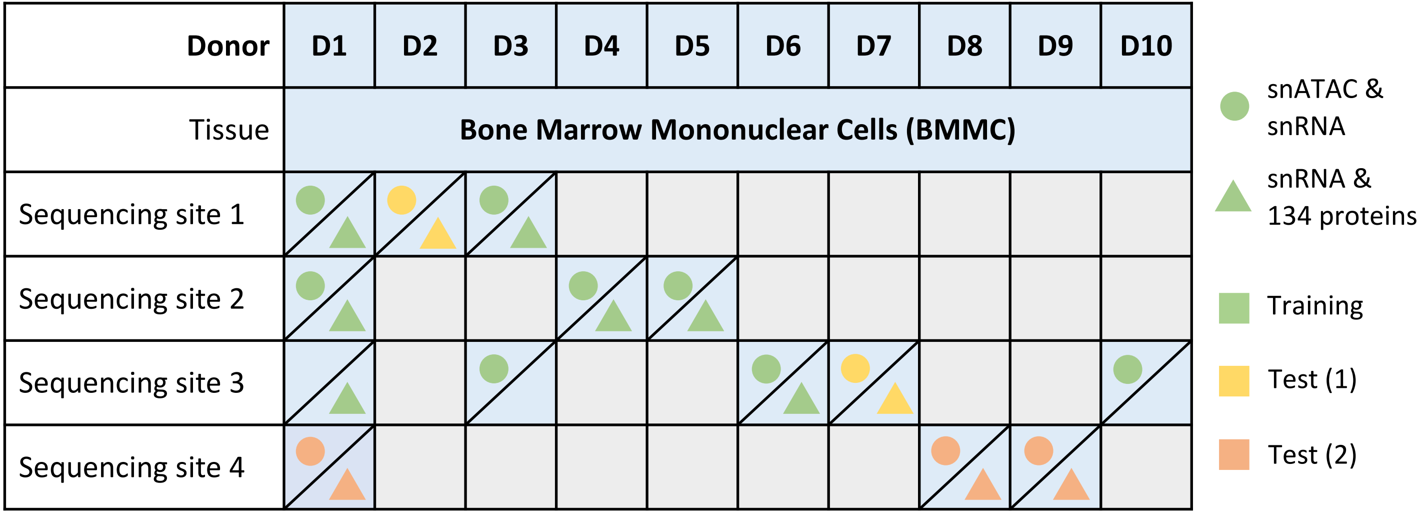

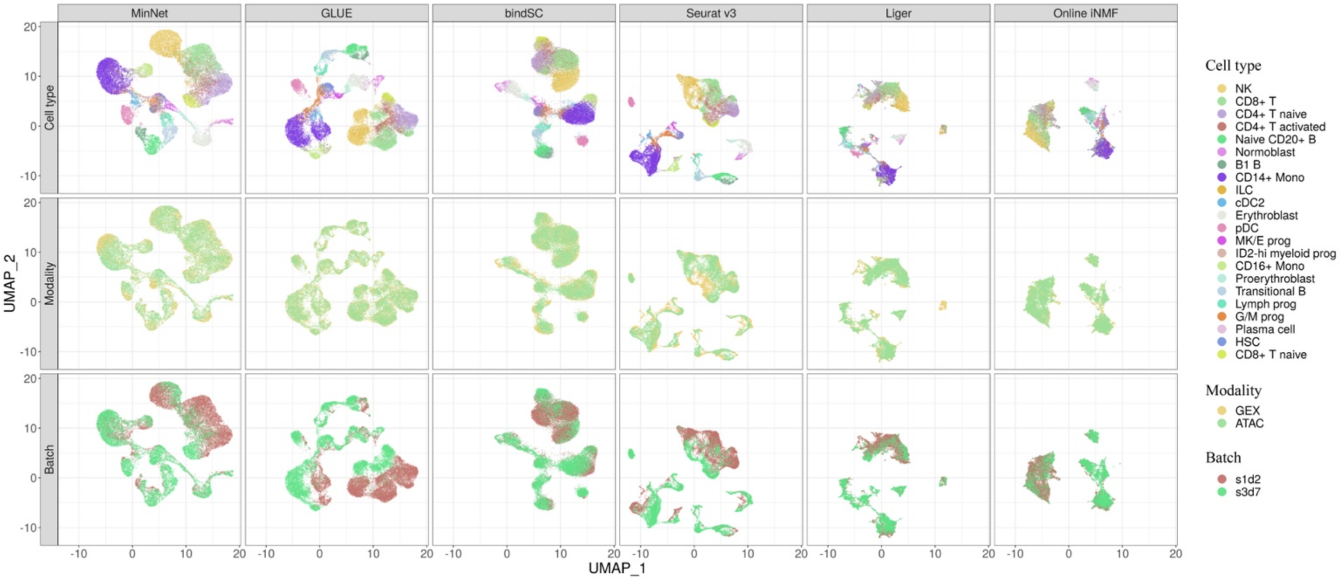

Figure S1. 10X Multiome BMMC validation set UMAP visualization of benchmarking algorithms labeled with cell type, modality, and batch.

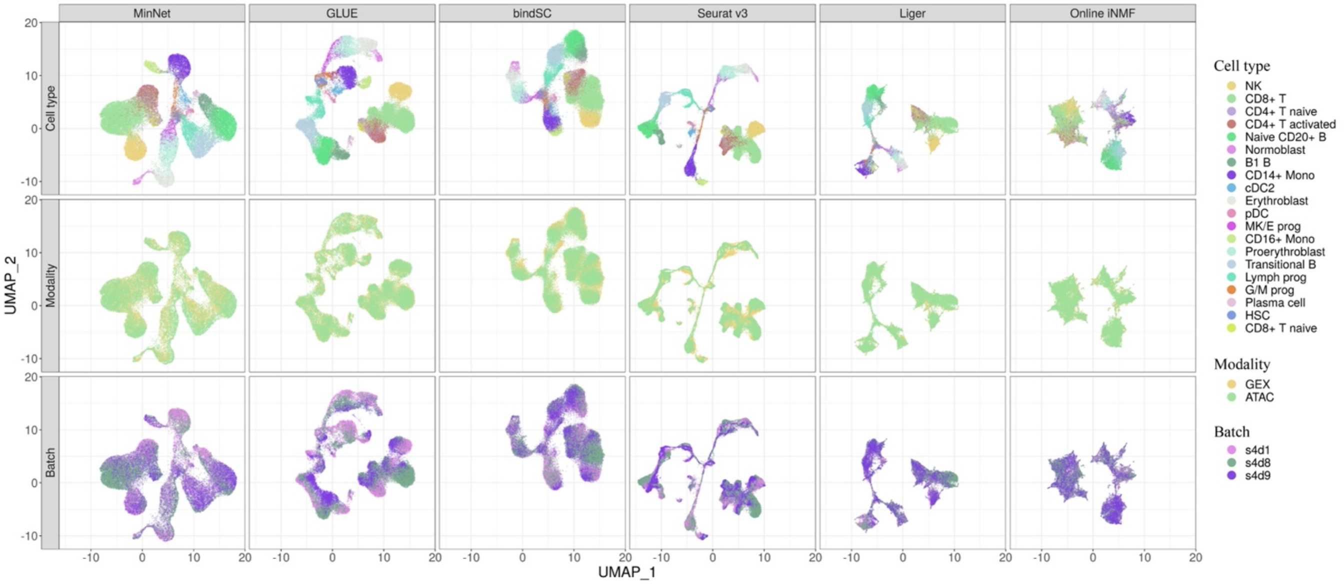

Figure S2. 10X Multiome BMMC test set UMAP visualization of benchmarking algorithms labeled with cell type, modality, and batch.

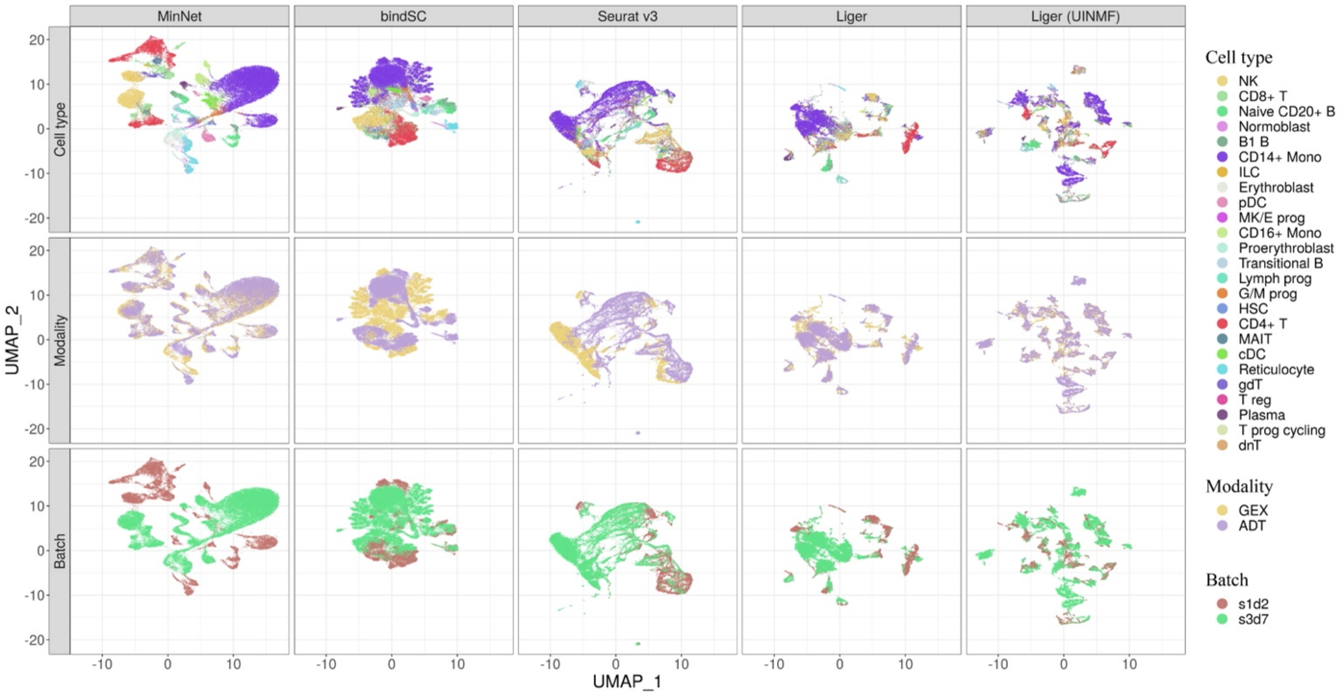

Figure S3. Cite-Seq BMMC validation set UMAP visualization of benchmarking algorithms labeled with cell type, modality, and batch

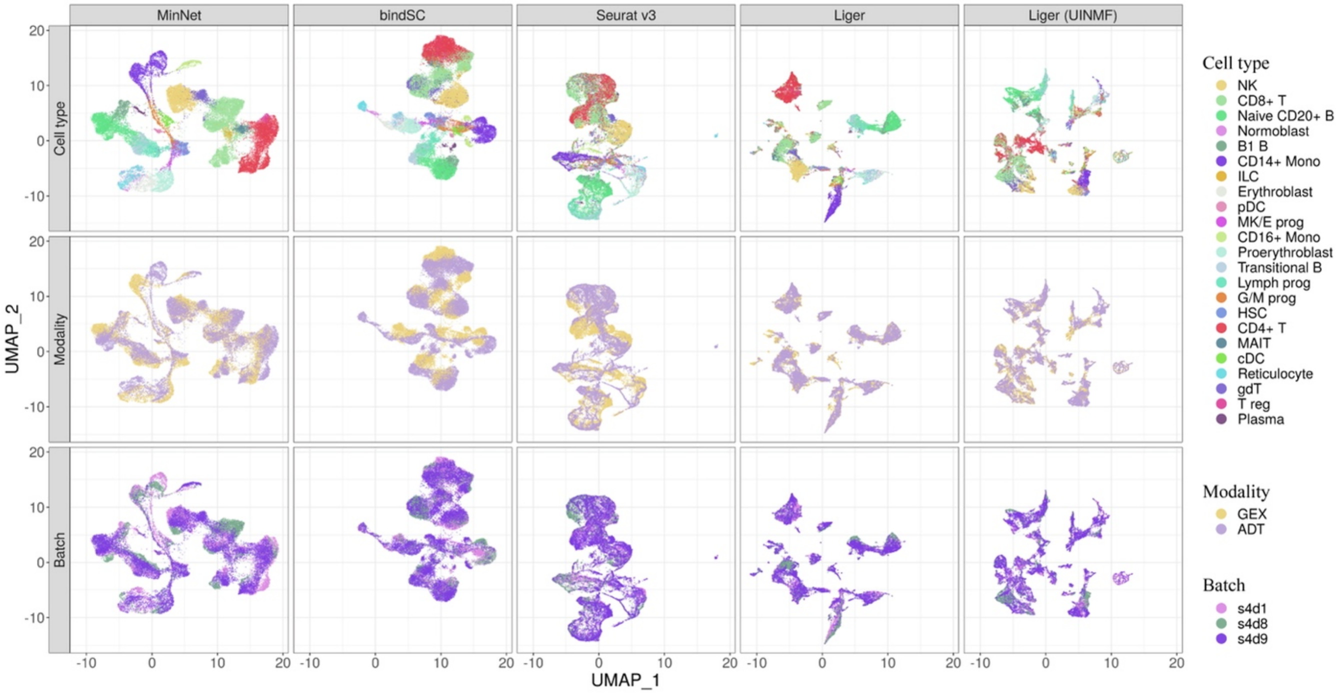

Figure S4. Cite-Seq BMMC test set UMAP visualization of benchmarking algorithms labeled with cell type, modality, and batch.

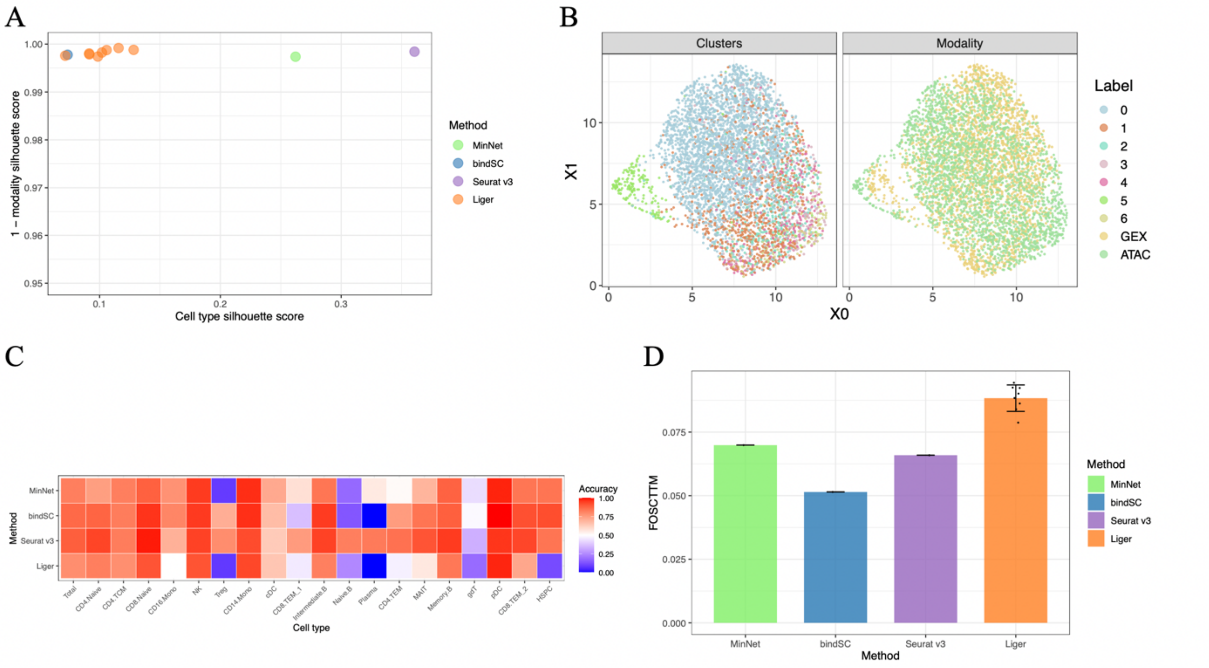

Figure S5. Model Generalizability Evaluation in external datasets. **A** Silhouette scores on the embedding space generated by all algorithms with 10X Multiome PBMC data. **B** UMAP visualization of 10X Multiome human brain data. **C** Label transfer accuracy of the PBMC data. **D** FOSCTTM score of algorithms with the PBMC data. We failed to replicate the results from GLUE’s paper, please refer to the performance mentioned in their paper using the same dataset.

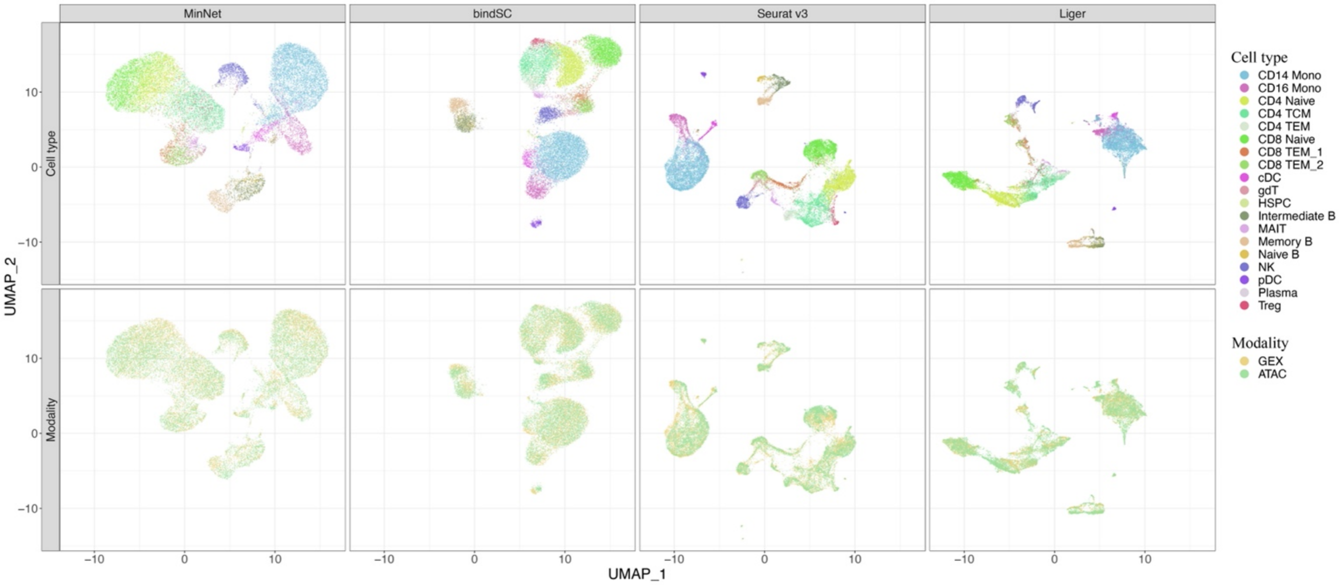

Figure S6. 10X Multiome PBMC dataset UMAP visualization of the co-embedding space labeled by cell type, modality, and batch.

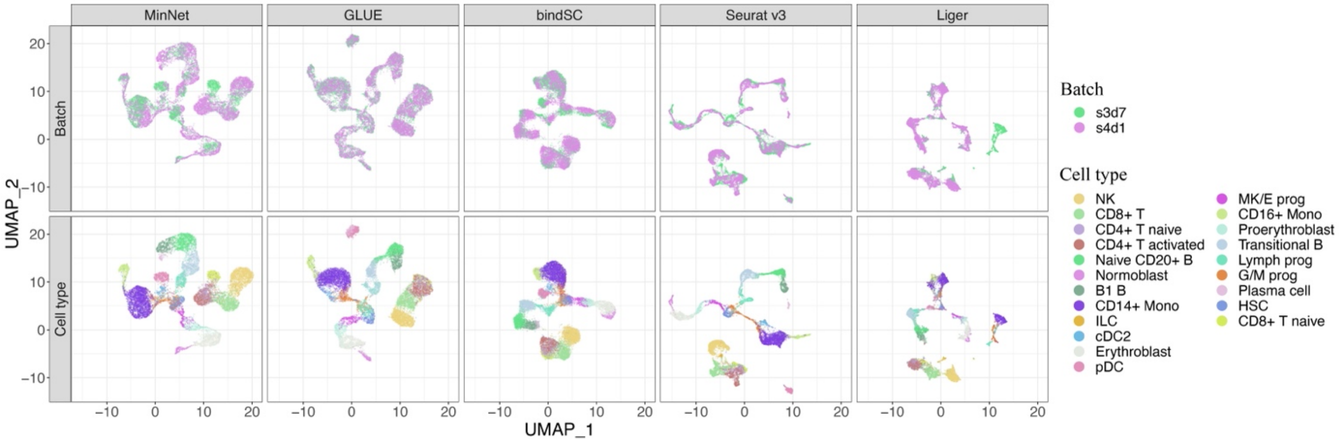

Figure S7. UMAP visualization of the co-embedding space labeled by cell type and batch in batch effect removal scenario 2. GEX data is from batch s3d7 and ATAC data is from batch s4d1.

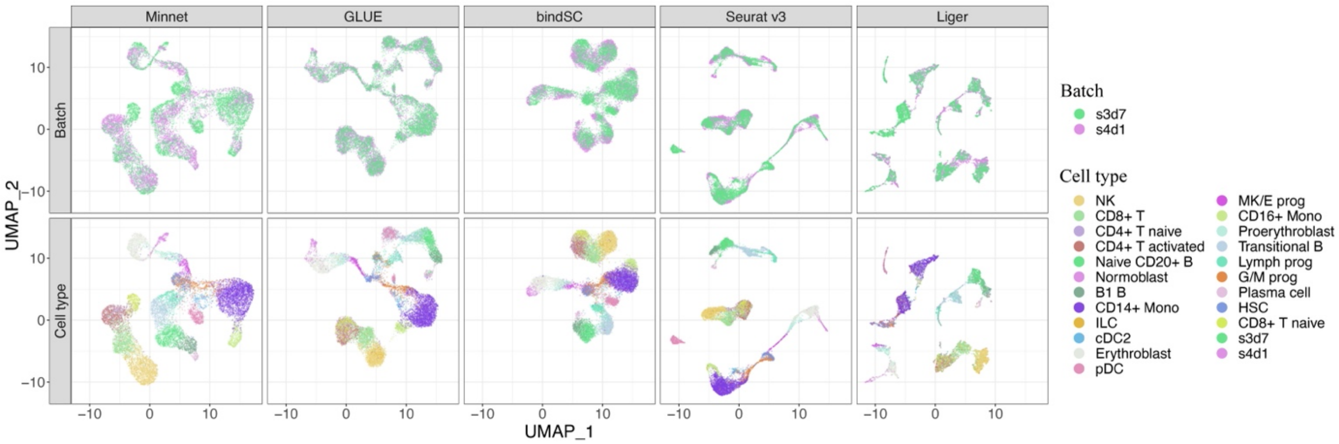

Figure S8. UMAP visualization of the co-embedding space labeled by cell type and batch in batch effect removal scenario 3. GEX data is from batch s4d1 and ATAC data is from batch s3d7.

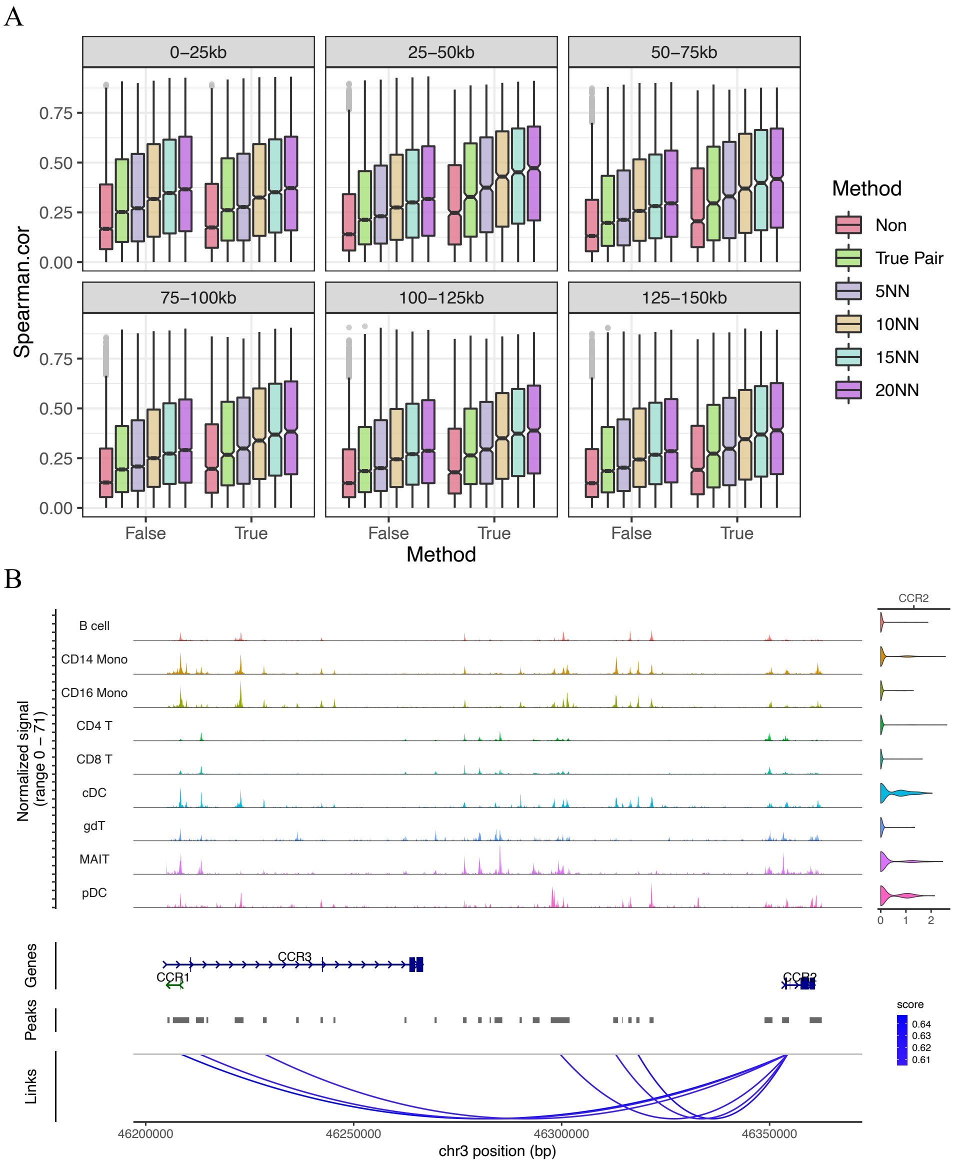

Figure S9. A Boxplot showing all correlation scores between gene-peak pairs grouped by distances and mini-bulk data. **B** Genome track for CCR2 and its 6 highly correlated peaks.

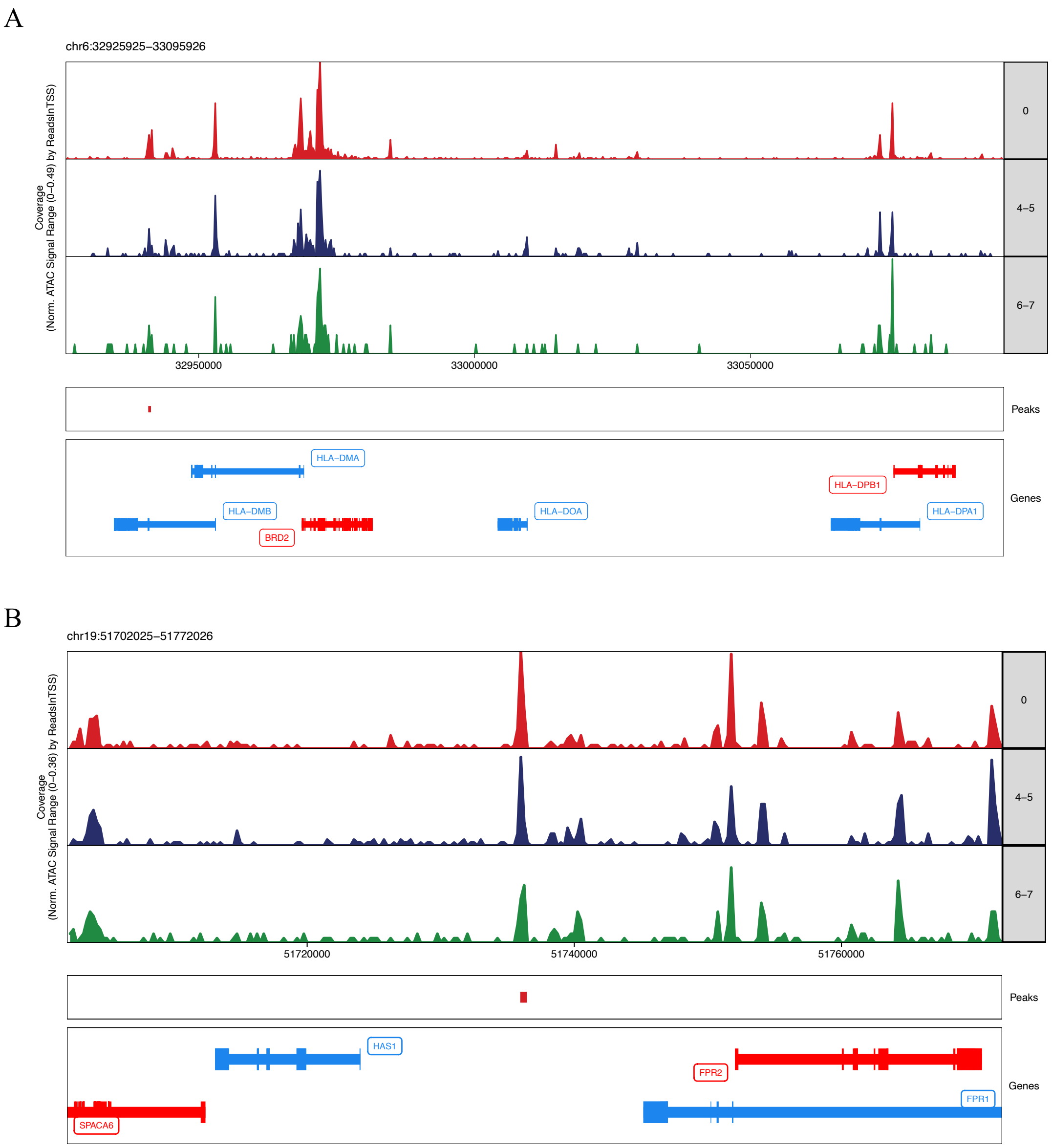

Figure S10. Genome tracks of examples mentioned in COVID-19 data analysis. **A** HLA-DPB1 and its associated peak (labeled in the peaks annotation row) in NK cells. **B** FPR2 and its associated peak in Monocytes.
